# Effects of surgical resection on tissue autofluorescence lifetime signatures in head and neck cancer: implications for intraoperative tumor margin assessment

**DOI:** 10.1117/1.JBO.31.9.096002

**Published:** 2026-09-04

**Authors:** Farzad Fereidouni, Willy Ju, Sarah Rezapourdamanab, Dena Sayrafi, Mohamed Abul Hassan, Brent Weyers, Xiangnan Zhou, Julien Bec, Dorina Gui, Andrew Birkeland, Laura Marcu

**Affiliations:** a Emory University, Department of Pathology and Laboratory Medicine, Atlanta, Georgia, United States; b University of California, Davis, Department of Biomedical Engineering, Davis, California, United States; c University of California, Davis, Department of Pathology and Laboratory Medicine, Davis, California, United States; d University of California, Davis, School of Medicine, Department of Otolaryngology Head and Neck Surgery, Davis, California, United States; e University of California, Davis, Department of Neurological Surgery, Department of Otolaryngology Head and Neck Surgery, Davis, California, United States

**Keywords:** head and neck cancer, intraoperative margin assessment, label-free imaging

## Abstract

**Significance:**

Accurate identification of residual tumor during head and neck cancer surgery is essential for preventing local recurrences and improving patient outcomes, yet current methods for intraoperative margin assessment remain limited. Fluorescence lifetime imaging (FLIm), a label-free optical technique, has shown strong potential for distinguishing cancerous from noncancerous tissue *in vivo*. However, the effect of tissue resection on autofluorescence properties is not well defined, raising the question of whether diagnostic signatures established *in vivo* translate reliably to *ex vivo* specimens.

**Aim:**

The aim is to characterize how fluorescence lifetime properties change between *in vivo* and *ex vivo* conditions across multiple head and neck anatomical sites and to assess how these changes affect diagnostic separability between cancerous and noncancerous tissue.

**Approach:**

A custom fiber-based, point-scanning FLIm system with phasor-based analysis was used to characterize fluorescence lifetime features *in vivo* and *ex vivo* across oral tongue, base of the tongue, and palatine tonsil tissue from 75 patients undergoing oropharyngeal surgery. A separability index based on phasor distributions was used to quantify cancer versus noncancer contrast across multiple spectral channels.

**Results:**

Resection-induced changes in decay dynamics and fluorescence lifetime values varied by anatomy: oral tongue and tonsil showed reduced contrast *ex vivo*, whereas the base of tongue demonstrated improved separation after excision. The base of the tongue showed a higher separability index *ex vivo* (0.43 versus 0.15 *in vivo*), whereas oral tongue and tonsil maintained superior contrast *in vivo* (0.27 versus 0.18 and 0.46 versus 0.28, respectively).

**Conclusions:**

Surgical resection substantially alters autofluorescence signatures in an anatomy-dependent manner, emphasizing the need to train and validate diagnostic algorithms independently for *in vivo* surgical guidance and *ex vivo* pathology assessment.

## 1 Introduction

Surgery, often combined with radiotherapy or chemotherapy, remains the primary curative intervention for many solid malignancies, including head and neck cancers.^1^ However, ensuring complete removal of all tumor cells remains a major clinical challenge, and the presence of residual malignant cells at the surgical margin is a well-established predictor of local recurrence and overall survival.^2^^,^^3^ Achieving clear histopathological margins is particularly difficult in the head and neck region, where tumors often arise in close proximity to critical anatomical structures.^4^ Recent evidence suggests that inadequate excision at primary surgery, even if followed by re-excision, is associated with higher recurrence rates and poor prognosis, highlighting the need for reliable intraoperative margin assessment.^5^^,^^6^

Recent advances in label-free imaging technologies have shown potential for intraoperative assessment of surgical margins both *in vivo* and *ex vivo*. These technologies include intraoperative fluorescence-guided surgery,^7^ ultrasound,^8^ optical coherence tomography,^9^^,^^10^ spectral imaging, and fluorescence lifetime imaging (FLIm).^11^ Among emerging label-free optical techniques, FLIm has shown strong potential for distinguishing cancerous from noncancerous tissue *in vivo* and advancing intraoperative tissue characterization.^12^^–^^14^ FLIm is a technique that can identify enhanced metabolic rates, which are often found with cancer cells and can promote a hypoxic environment.^15^ These lead to changes in endogenous fluorescent molecules such as Nicotinamide adenine dinucleotide (NADH) and flavin adenine dinucleotide (FAD) molecular configuration which can provide significant information on metabolic changes associated with the malignant (and possibly premalignant) state.^16^^–^^18^ Other metabolism-associated factors that can be measured using FLIm include hemoglobin oxygenation levels near a tumor, which decrease as the growing tumor extracts more oxygen from the blood.^19^^,^^20^ In addition, poor perfusion in the tumor environment leads to the retention of waste products, decreasing the local pH.^21^ Changes in the extracellular matrix, particularly in collagen structure and crosslinking during oncogenic transformation, also contribute to variations in autofluorescence, offering further contrast that FLIm can leverage for tissue characterization.^22^^,^^23^ In previous studies,^11^^,^^12^^,^^14^ we demonstrated that changes in these factors can be measured using FLIm and implemented in an intraoperative *in vivo* setting. However, it remains unclear whether fluorescence lifetime signatures acquired *in vivo* are preserved and can be reliably reproduced in *ex vivo* conditions. This distinction is clinically critical, as intraoperative decision-making relies on*in vivo* imaging, whereas validation and downstream pathology workflows are typically performed on*ex vivo* specimens. Here, we investigate how FLIm features change following tissue excision, highlighting its potential implications for tumor margin assessment across both *in vivo* and*ex vivo* settings.

Tissue excision introduces rapid biochemical and metabolic changes, such as loss of perfusion and hypoxia, that may alter fluorescence lifetime measurements. Although FLIm imaging of excised tissue specimens *ex vivo* offers several practical advantages, further investigation is needed to determine whether the sensitivity and specificity demonstrated *in vivo* can be reliably reproduced in the *ex vivo* settings. This study was designed to investigate these changes and assess the differences between *in vivo* and *ex vivo* FLIm imaging on the same tissue samples. Using the phasor approach to analyze fluorescence decay data, we evaluated the separability of normal tissue from squamous and invasive cell carcinoma for the oral tongue, tonsil, and base of tongue tissues from 75 patients. We performed a paired *in vivo–ex vivo* analysis using phasor-based FLIm across multiple anatomical sites to quantify how fluorescence lifetime features and diagnostic contrast evolve following tissue excision. Although the most recent data described in this study are primarily in the context of head and neck cancers, the methodologies and the insights gained could be generalized and employed for other tissue and organ types, broadening the applicability of FLIm to a wider range of cancers.

## 2 Materials and Methods

### 2.1 Setup

**Fig. 1:**
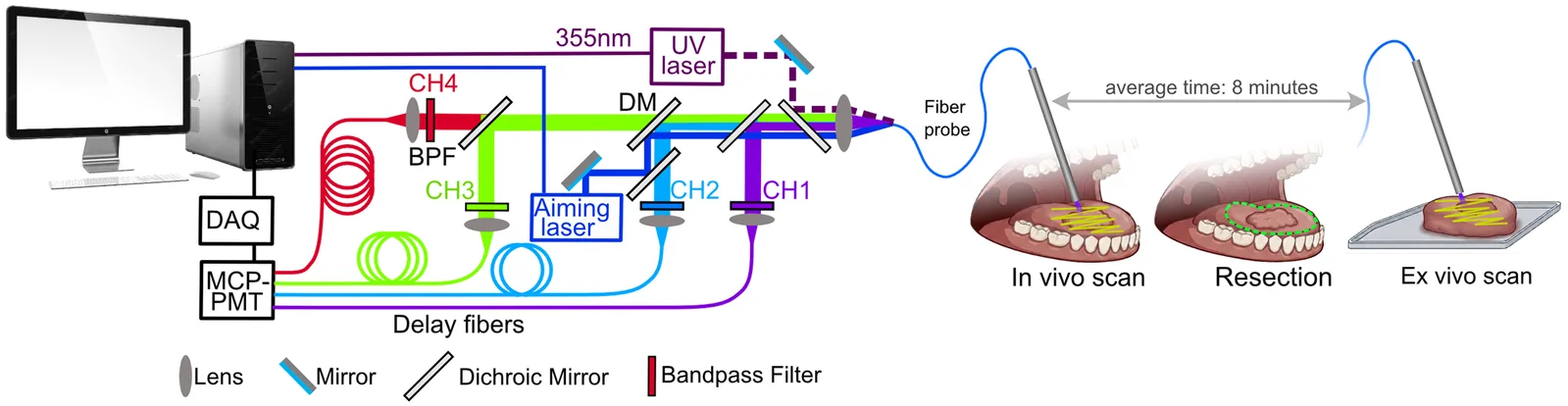
Schematic diagram of the FLIm instrument and experimental steps. A frequency-tripled Nd:YAG microchip laser (STV-02E-140, TEEM photonics, Meylan, France) with repetition rate of 120 Hz and pulse width of 600 ps was used as fluorescence excitation light source. Fluorescence excitation and emission were guided to and from the target by a single 365-μm core diameter all silica multimode optical fiber (FVP400440480, Polymicro). A wavelength selection module was used to separate fluorescence emission into three nonoverlapping spectral bands (CH1: 390±20  nm, CH2: 470±14  nm, CH3: 542±25  nm, and CH4: 629±26.5  nm). The spectral channels were temporally separated by passing the emission pulse through three delay fibers (FVP400440480, Polymicro) of different lengths. A single microchannel plate photomultiplier tube (R3809U-50, Hamamatsu, Japan) detector was used to detect the temporally multiplexed signal. To time-resolve the fluorescence emission, the electrical pulses from the MCP-PMT were recorded by a 12.5  Gs/s, 8-bit digitizer (PXIe-5185, National Instruments, Debrecen, Hungary) with 1.5 GHz analog bandwidth and 80 ps temporal resolution, which was synchronized with the laser pulse. A 445-nm continuous-wave aiming laser was used to indicate the location of the FLIm point measurements. Right panel: FLIm measurements were first acquired from the external tissue surface *in vivo*. Following surgical resection and specimen transfer, the corresponding external surface was measured ex vivo, with an average interval of ∼8  min between the *in vivo* and *ex vivo* acquisitions. Abbreviations: FLIm, fluorescence lifetime imaging; Nd:YAG, neodymium-doped yttrium aluminum garnet; CH, channel; MCP-PMT, microchannel plate photomultiplier tube; DAQ, data acquisition; UV, ultraviolet.

A custom-designed, fiber-based point-scanning FLIm instrument was employed for data collection in this study. Detailed information on the hardware^24^ and data processing^25^ for FLIm can be found in our prior publications with a schematic diagram of the FLIm system shown in Fig. 1. In brief, the FLIm system utilized a 365  μm core multimode optical fiber to deliver pulsed UV laser excitation light at 355 nm (600 ps, 2  μJ) with a repetition rate of 120 Hz, generating autofluorescence from a small region of the tissue under examination ($∼1\;{\mathrm{mm}}^{2}$). Tissue autofluorescence is collected with the same fiber optic probe and separated into four distinct spectral bands (CH1: 390±20  nm, CH2: 470±14  nm, CH3: 542±25  nm, and CH4: 629±26.5  nm) utilizing dichroic mirrors and bandpass filters.^26^ These spectral bands corresponded to the autofluorescence emission peaks of collagen, NAD(P)H, FAD, and porphyrins. These spectral bands were selected to capture key metabolic and structural fluorophores relevant to tissue differentiation. In this study, Channel 4 was excluded from the analysis because of its low signal-to-noise ratio (SNR), which could reduce the reliability of the lifetime estimates and introduce greater uncertainty into the results.

The optical signals from each spectral channel were time-multiplexed onto a single microchannel plate photomultiplier tube (R3809U-50, Hamamatsu, Japan), then amplified and time-resolved by a high-speed digitizer at 80 ps intervals (PXIe-5185, National Instruments, Austin, Texas, United States). In addition, a separate continuous-wave diode laser emitting at 445 nm (TECBL50G-440-USB, World Star Tech, Canada) was used to highlight the location of FLIm point measurements.^26^

FLIm data were acquired intraoperatively in two distinct surgical scenarios: (1) Transoral robotic surgical procedures (TORS) employing the da Vinci SP robotic system (Intuitive Surgical, Inc), typically for oropharyngeal cancer cases, and (2) nonTORS procedures conducted manually, often for oral cavity cancer. In nonTORS procedures, a handheld fiber probe (Omniguide Laser Handpiece) and an endoscopic camera (Stryker) were combined, whereas in TORS procedures, the fiber optic probe was manipulated using robotic instruments, with the surgical field being visualized through the integrated da Vinci camera. For both *in vivo* and *ex vivo* measurements, FLIm scanning was performed on the epithelial surface of the tissue. Depending on the surgical context, the FLIm surface scanning required ∼1 to 2 min for both *in vivo* imaging prior to specimen excision and *ex vivo* imaging after resection. The average interval between the corresponding *in vivo* and *ex vivo* measurements was ∼8  min. Rather than being experimentally predetermined, this interval reflected the typical surgical workflow, including specimen resection, handling, and transfer for *ex vivo* imaging. To ensure comparability, imaging conditions were standardized between the two measurements, including probe contact, angle, and illumination settings.

### 2.2 Data Analysis

**Fig. 2:**
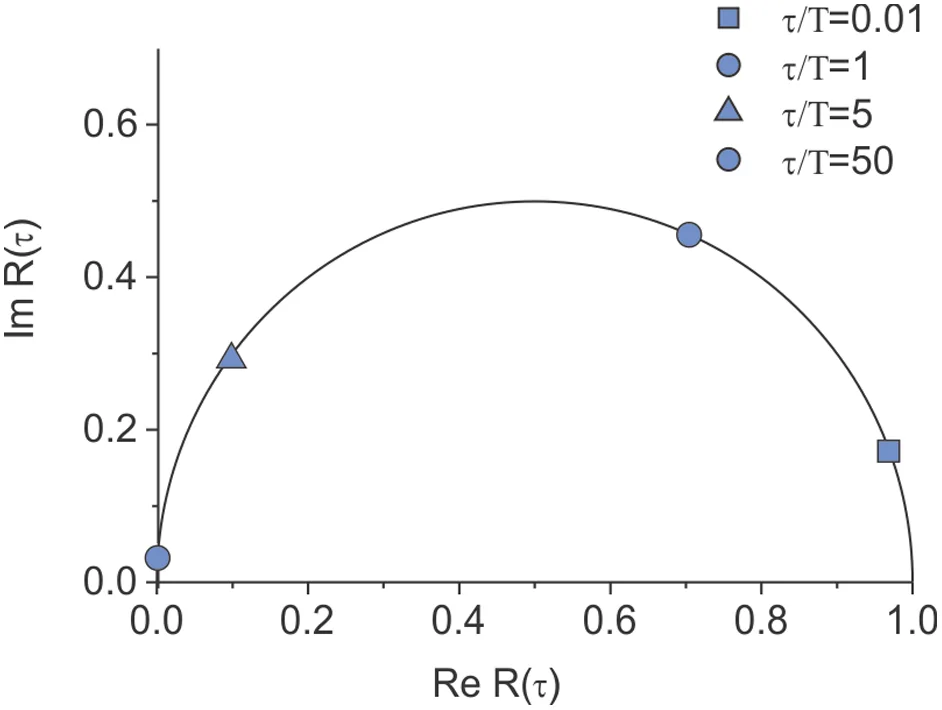
Plot of semicircle ImR(τ) versus ReR(τ). The curve shows all possible single component lifetimes from τ/T=0.01 to τ/T=50 at ω=2π/T.

FLIm data were analyzed using a phasor approach previously reported^27^^,^^28^ Briefly, the first harmonics of the Fourier transformation of decay curves are plotted on a 2D plot where the horizontal axis is the real part of the transformation, and the vertical axis is the imaginary part of the transformation. As shown in Fig. 2, all the single exponential decays fall on a semicircle where the shortest lifetimes are at the coordinate of (1,0), and the longest lifetimes are mapped at the coordinate of (0,0). The phasor plot data points and the image pixels are correlated; every point in the phasor plot can be traced back to pixels with the same spectral or lifetime property on the image. Moreover, every decay or spectrum is mapped onto a unique position in the phasor plot, and the position of the phasor determines the lifetime or the spectrum. A region of interest in the phasor plot can be back-projected to the pixels correlated with the selected phasor points. This results in fast and convenient image segmentation.

The application of the phasor approach to time-domain data with different time gate settings and acquisition periods has been shown before.^27^ This theoretical framework can be applied to different time gate configurations. The general phasor semicircle is expressed by

$$R(\tau )=\frac{1}{\cos(\frac{\pi }{K})-\sin(\frac{\pi }{K})\text{coth}(\frac{T}{2K\tau })j},$$

where K is the number of sampling points, T is the digitized fluorescence-decay window used in the Fourier transform-based phasor calculation, and t is the lifetime. R is a complex number, and the reference semicircle is generated by drawing the imaginary part of R versus the real part. As the lifetime increases, the phasor moves on the semicircle from right to left.

In our experiments, T=32  ns and K=400. Here, T does not represent the laser repetition period or the overall data-acquisition duration. The fluorescence-decay waveform was analyzed at its native 80-ps sampling interval without temporal binning. Because K was large and the sampling interval was short relative to the measured fluorescence lifetimes, the finite-sampling phasor curve closely approximated the standard phasor semicircle. Therefore, the standard phasor expression corresponding to the K→∞ limit was used, and no finite-gate correction was applied. When K→∞, R converges to the standard phasor curve:^28^

$$R(\tau )=\frac{1}{1-j\frac{2\pi }{T}\tau }.$$

Figure 2 shows the modified reference semicircle for a lifetime range from τ/T=0.01 to τ/T=5. The average lifetime can be estimated by the following equations:

$$\tau =\frac{T}{2\pi }\frac{\mathrm{ImR}(\tau )}{\mathrm{ReR}(\tau )}.$$

Data analysis was performed using a custom MATLAB script, and all data points were subjected to a filtering process to ensure quality by applying a 30 dB SNR threshold, whereby any data with an SNR below this value was excluded from analysis to ensure reliable lifetime estimation and exclude low-quality signals.

### 2.3 Separability Index

Average lifetime alone may not fully capture differences due to the multiexponential nature of fluorescence decay; therefore, a separability index based on phasor distributions was used. A separability index method previously utilized to estimate lifetime values from a bi-exponential decay curve^29^ was adapted to the current study:

$$S=\frac{\left|\tau_{1}-\tau_{2}\right|}{\sqrt{\mathrm{var}\left(\tau_{1}\right)+\mathrm{var}\left(\tau_{2}\right)}}.$$

Extending this approach to other quantities is easily achieved, where we use it to evaluate the separability of phasor clouds by measuring the average location of the clouds and their variances. For two phasor clouds indicated by $P_{1}$ and $P_{2}$ the separability index is calculated by

$$S=\frac{\left|\text{mean}(P_{1})-\text{mean}(P_{2})\right|}{\sqrt{\mathrm{var}\left(P_{1}\right)+\mathrm{var}\left(P_{2}\right)}}.$$

**Fig. 3:**
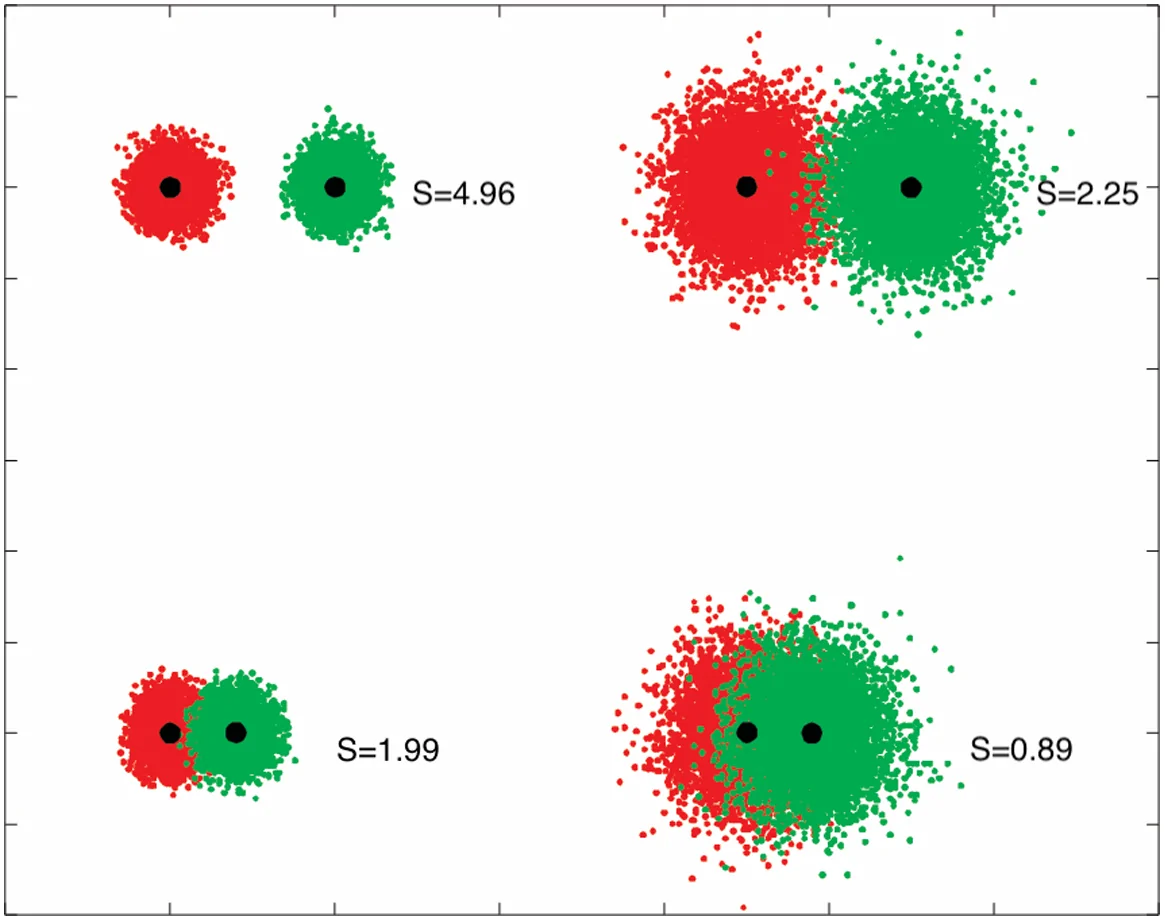
Separability index for clouds with the same mean values but different noise levels reflected in cloud shape.

Figure 3 shows different configurations of synthetic phasor clouds generated by Poisson noise and their corresponding separability index. It is worth noting that the separability is not just a measure of how distant two clouds are, but it also takes into account the size of the clouds and how much overlap they create.

### 2.4 Description of the Patient Cohort & Tumor Type

Eligible patients were recruited in the study over a five-year period from 2016 to 2021. The research was performed under the approval of the UC Davis Institutional Review Board (IRB) and with the patient’s informed consent. FLIm was performed on a total of 90 patients. Only patients with a newly diagnosed preoperative diagnosis of HNC of either the oral cavity or oropharynx were enrolled in this study. FLIm measurement locations were correlated with histopathology through visual landmarks, and only regions with reliable correspondence were included. FLIm data from 15 patients were excluded from the analysis for the following reasons. Data from eight patients were unable to be analyzed due to either accurate registration with tissue histopathology or instrumentation issues (e.g., malfunctioning software or damaged fiber optic probe) during the procedure. Data from seven additional patients with tumors presenting limited- to no- mucosal involvement (i.e., the extent of tumor comprised only the lamina propria or deeper into the submucosa, see limitation section).

Accordingly, data from N=75 patients with distinct anatomies (Table 1) were used for classifier training and testing. Tissue labels (cancerous versus non-cancerous) were assigned based on histopathological evaluation by expert pathologists. Each patient contributed one surgical specimen. Most specimens contained areas classified as cancerous or noncancerous, while some contained only one tissue type, as determined by histopathological evaluation. The "other" anatomy label included glossotonsillar sulcus, pharynx, floor of the mouth, lip, retromolar trigone, gingivae, palate, and vallecula. Among these patients, N=40 patients presented with HPV mediated oropharyngeal cancer confirmed by immunohistochemistry. Where p16+ is used as nomenclature for HPV-mediated tumor and p16- for tumor uninvolved with HPV.

**Table 1:** Summary of FLIm data acquisition across tissue types and anatomical sites in head and neck cancer patients. Number of FLIm data points collected *in vivo* and *ex vivo*, grouped by tissue type (cancerous versus noncancerous) and anatomical location. Total number of patients contributing to each category is also indicated. Average points per specimen were calculated by dividing the total number of FLIm points by the number of specimens in the corresponding category.

Anatomy	Tissue Label	No. Patients (N)	FLIm Points *In Vivo* (n)	Average *In Vivo* Points/Specimen	FLIm Points *Ex Vivo* (n)	Average *Ex Vivo* Points/Specimen
*Palatine Tonsil*	*Cancerous*	27	22,874	847.2	24,454	905.7
*Palatine Tonsil*	*Noncancerous*	31	28,866	931.2	18,561	598.7
*Oral Tongue*	*Cancerous*	25	18,053	722.1	30,274	1211
*Oral Tongue*	*Noncancerous*	25	12,966	518.6	21,620	864.8
*Base of the Tongue*	*Cancerous*	14	5797	414.1	9550	682.1
*Base of the Tongue*	*Noncancerous*	19	22,170	1,166.8	10,971	577.4

## 3 Results

### 3.1 Evaluation of *In Vivo* versus *Ex Vivo* FLIm Measurements (Case Study)

**Fig. 4:**
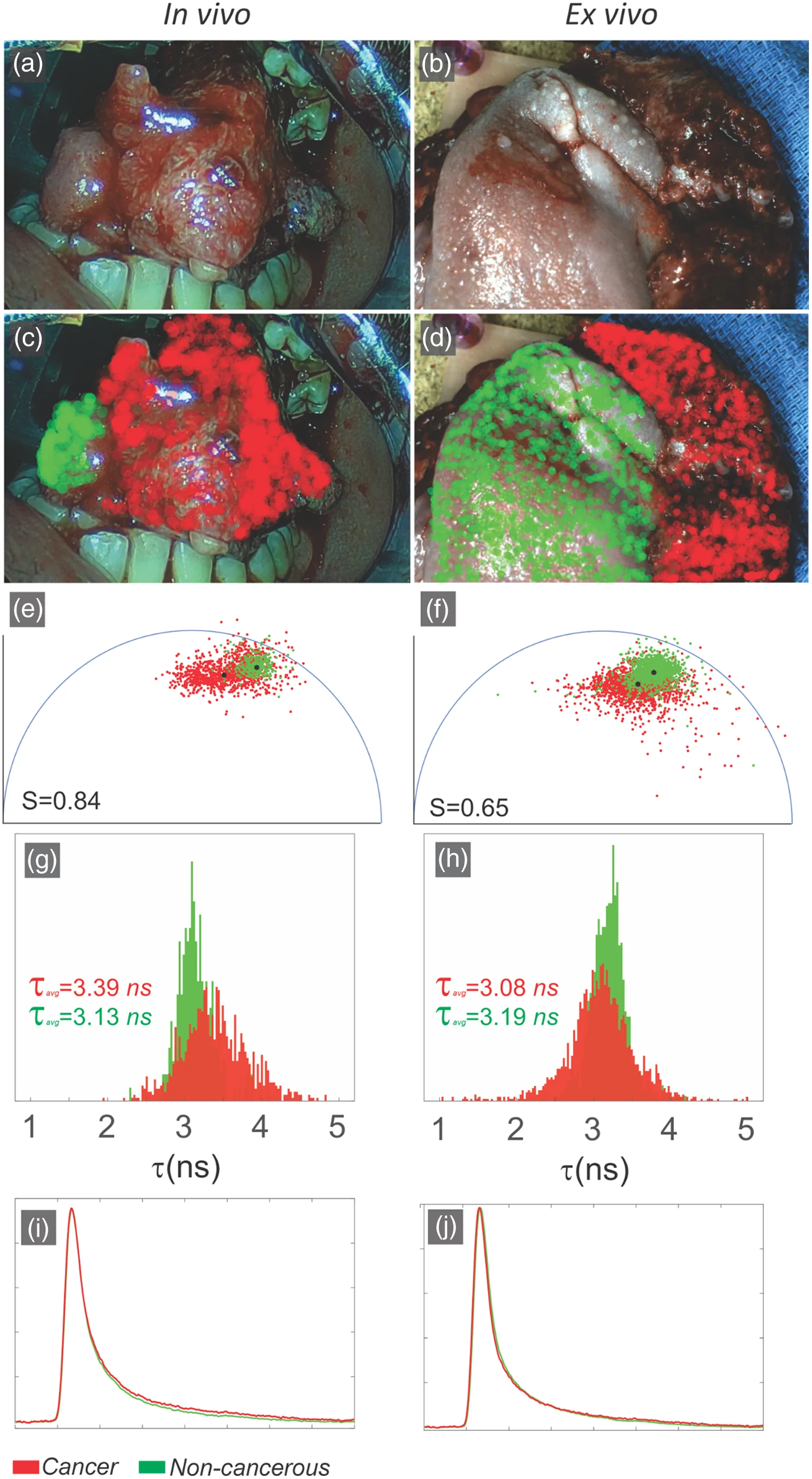
Demonstration of tissue transitioning from *in vivo* to *ex vivo*, showing the superior tongue under white light. (a) *In vivo* and (b) *ex vivo* of a 57-year-old male patient. Overlay of FLIm measurement points are color-coded by green and red for healthy and cancerous tissue in (c) *in vivo* and (d) *ex vivo* setting. FLIm data points are plotted on phasor plots for (e) *in vivo* and (f) *ex vivo* measurements. The separability between cancerous and noncancerous tissue decreases following tissue resection. Histograms (g) *in vivo* and (h) *ex vivo* and fluorescence decay curves (i) *in vivo* and (j) *ex vivo* are demonstrated. The profile for the histograms demonstrates a clear shift going from *in vivo* to *ex vivo.*

Figure 4 depicts white light images capturing the oral tongue before [Fig. 4(a)] and after [Fig. 4(b)] excision in a 68-year-old male patient. The FLIm measurement data points are overlaid on the images and color-coded according to cancerous versus noncancerous groups, with green representing noncancerous tissue and red indicating cancerous regions [Figs. 4(c) and 4(d)]. Phasor plots in *in vivo* [Fig. 4(e)] and *ex vivo* [Fig. 4(f)] conditions illustrate the data points for these cancerous (red) and noncancerous (green) regions. Phasor plots reveal reduced separability between cancerous and noncancerous tissue in the*ex vivo* (S=0.65) setting compared to the *in vivo* (S=0.84) condition.

Histograms of fluorescence lifetime values [Figs. 4(g) and 4(h)] demonstrate a shift toward shorter lifetimes for cancerous tissue in the *ex vivo* condition (τ(c):3.39  ns→3.08  ns), while noncancerous tissue shows minimal change (τ(nc):3.13  ns→3.19  ns). In addition, the *ex vivo* distributions appear broader, indicating increased heterogeneity following tissue excision. Fluorescence decay curves [Figs. 4(i) and 4(j)] further demonstrate altered decay dynamics after resection, consistent with the observed shifts in phasor distributions and reduced distinction between tissue types.

### 3.2 *In Vivo* versus *Ex Vivo* Lifetime Comparison Across Tissue Types and Emission Wavelengths

**Fig. 5:**
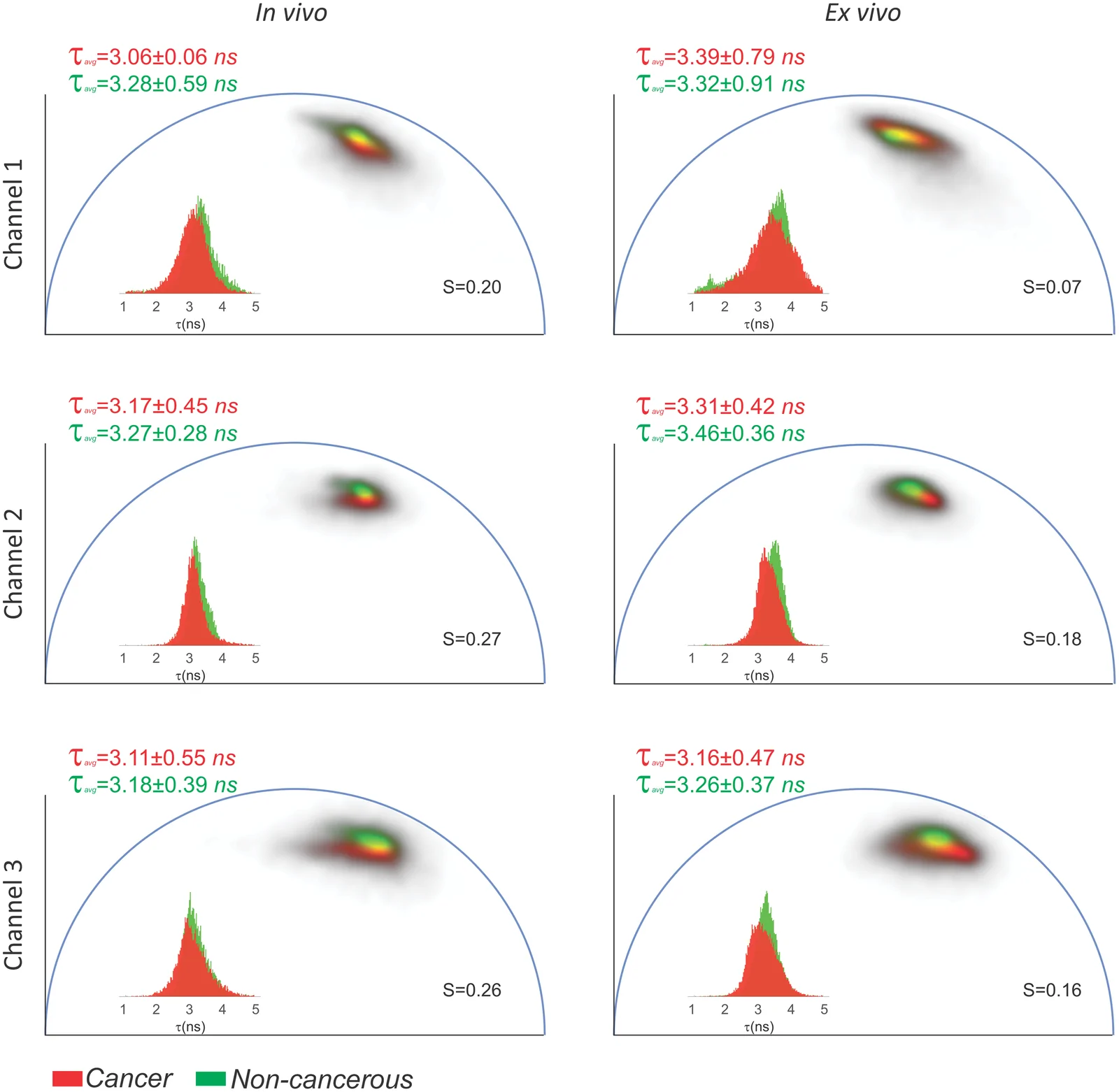
Phasor plots and histograms of average lifetimes for *in vivo* and *ex vivo* FLIm measurements from oral tongue tissue highlight differences between noncancerous and cancer tissues, labeled green and red, respectively. The separability index (S) is shown next to each plot, with higher values indicating improved separability between cancer and noncancerous tissue.

To assess the generalizability of these findings beyond a single instance, we expanded the analysis to the entire patient cohort (N=75). Figure 5 shows the results for oral tongue tissue samples (N=25 patients). Analysis of the average lifetimes shows that both cancerous and noncancerous tissues exhibited an overall increase in lifetime values from *in vivo* to *ex vivo* conditions across all spectral channels.

In channel 1, both cancerous and noncancerous tissues show a shift toward longer lifetimes. The difference between mean lifetimes decreases (Δτ:0.22  ns→0.07  ns), and increased overlap between the phasor clouds results in reduced separability (S: 0.20→0.07). In channel 2, a similar trend is observed, where both tissue types shift toward longer lifetimes, with noncancerous tissue maintaining higher lifetime values. Although the difference between mean lifetimes increases slightly (Δτ:0.10  ns→0.15  ns), increased overlap between the phasor clouds in the *ex vivo* condition results in reduced separability (S: 0.27→0.18). In channel 3, lifetime values also increase for both cancerous and noncancerous tissues, with a slight increase in mean lifetime difference (Δτ:0.07  ns→0.10  ns), but broader distributions and increased overlap in the *ex vivo* condition led to reduced separability (S: 0.26→0.16). Across all channels, lifetime histograms demonstrate a shift toward longer lifetimes in the *ex vivo* condition along with broader distributions and increased overlap, indicating increased heterogeneity following tissue excision. Overall, oral tongue tissue shows increased lifetimes with reduced separability in both tissue types.

**Fig. 6:**
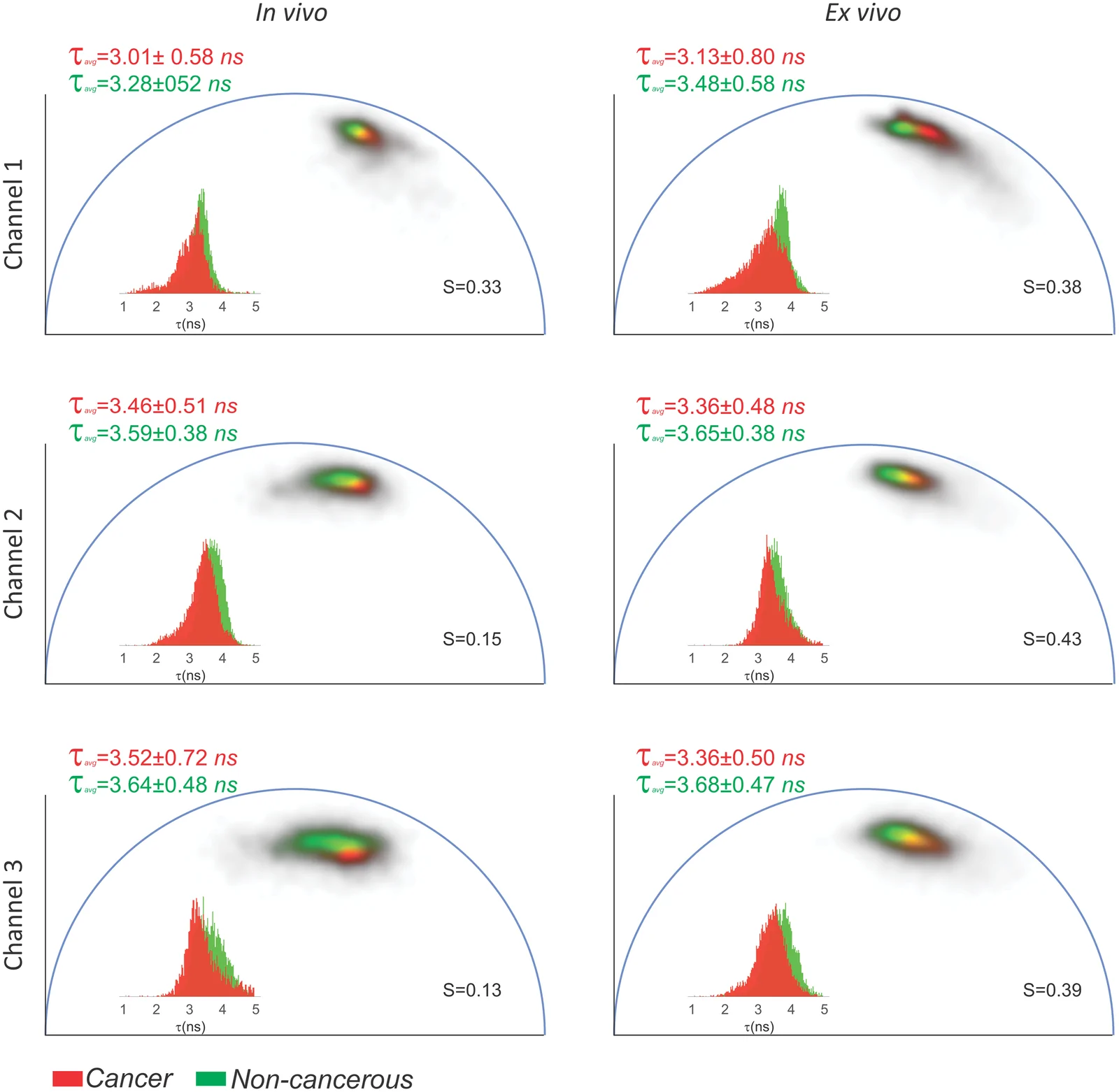
Phasor plots and histograms of average lifetimes for *in vivo* and ex vivo FLIm measurements from the base of the tongue tissue highlight differences between noncancerous and cancer tissues, labeled green, and red, respectively. The separability index (S) is shown next to each plot, with higher values indicating improved separability between cancer and noncancerous tissue.

Figure 6 shows the results for the base of tongue tissue samples (N=19 patients). In this group, the behavior varies across channels. In channel 1, for both cancerous and noncancerous tissues, average lifetime increases when transitioning from *in vivo* to *ex vivo*. In contrast, in channels 2 and 3, the lifetime for cancerous tissue decreases while noncancerous tissue shows an increase in lifetime, resulting in opposing trends between the two tissue types. This behavior contrasts with the oral tongue, where both tissue types demonstrated an overall increase in lifetime *ex vivo*. In channel 1, both tissue types show an increase in lifetime (3.01  ns→3.13  ns and 3.28  ns→3.48  ns), with a corresponding increase in separability (S: 0.33→0.38). In channel 2, lifetime in cancerous tissue decreases (3.46  ns→3.36  ns) while noncancerous tissue shows increase (3.59  ns→3.65  ns), leading to a larger separation between the distributions and a substantial increase in separability (S: 0.15→0.43). In channel 3, a similar opposing trend is observed, with lifetime for cancerous tissue decreasing (3.52  ns→3.36  ns) and noncancerous tissue increasing (3.64  ns→3.68  ns), resulting in enhanced separation and increased separability (S: 0.13→0.39).

These opposing trends between cancerous and noncancerous tissues in the base of the tongue increase the effective distance between the distributions while reducing overlap, consistent with the observed increase in separability from *in vivo* to *ex vivo* across all channels. Overall, the base of the tongue tissue exhibits opposing lifetime shifts between cancerous and noncancerous regions, resulting in enhanced diagnostic separability in the ex vivo condition.

**Fig. 7:**
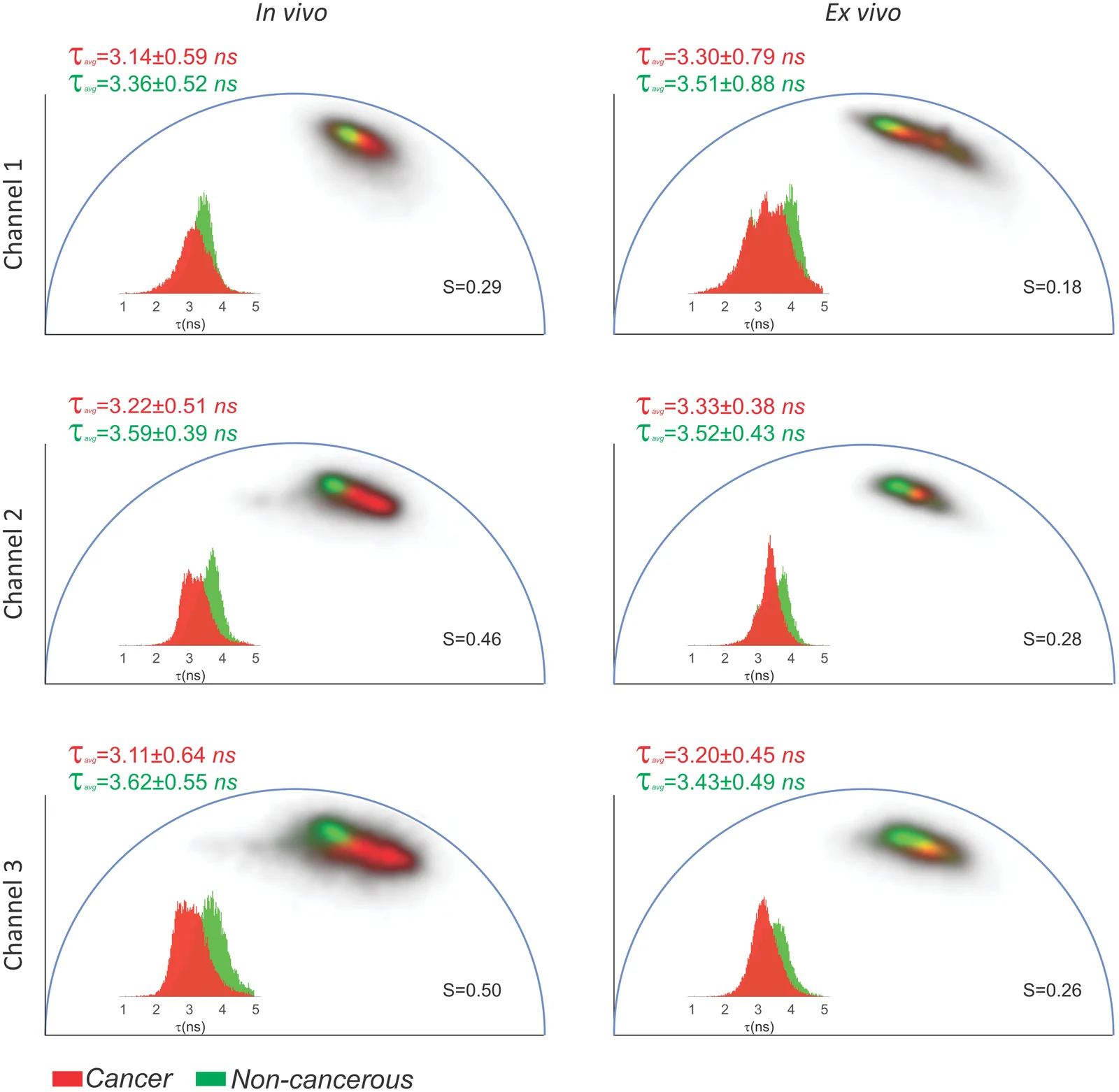
Phasor plots and histograms of average lifetimes for *in vivo* and *ex vivo* FLIm measurements from tonsil tissue highlight differences between noncancerous and cancer tissues, labeled green and red, respectively. The separability index (S) is shown next to each plot, with higher values indicating improved separability between cancer and noncancerous tissue.

Figure 7 shows the results for the palatine tonsil tissue samples (N=31 patients), where the behavior differs from the oral tongue and the base of the tongue. In channel 1, both cancerous and noncancerous tissues show increase in lifetime when transitioning from *in vivo* to *ex vivo* (3.14  ns→3.30  ns and 3.36  ns→3.51  ns, respectively), accompanied by increased overlap and a decrease in separability (S: 0.29→0.18). In channel 2, the lifetime for cancerous tissue increases (3.22  ns→3.33  ns) while noncancerous tissue shows decrease in lifetime (3.59  ns→3.52  ns), resulting in opposing trends; however, increased overlap between the distributions leads to reduced separability (S: 0.46→0.28). In channel 3, a similar opposing pattern is observed, with cancerous tissue showing increase in lifetime (3.11  ns→3.20  ns) and noncancerous tissue decreasing in lifetime (3.62  ns→3.43  ns), again resulting in increased overlap and a reduction in separability (S: 0.50→0.26).

Despite these varying trends in lifetime changes across channels, both the phasor distributions and corresponding histograms demonstrate greater overlap in the *ex vivo* condition. Notably, palatine tonsil tissue exhibits the highest separability under *in vivo* conditions (S=0.50 in channel 3) among all tissue types. Overall, increased distribution overlap in the ex vivo condition leads to reduced diagnostic separability in tonsil tissue.

Across all tissue types, separability is associated with the relative distance and overlap between cancerous and noncancerous lifetime distributions. In the oral tongue tissue, both tissue types shift in the same direction, accompanied by increased overlap and reduced separability. In the base of the tongue tissue, opposing lifetime shifts between cancerous and noncancerous regions are associated with increased separation and higher separability. In tonsil tissue, mixed and partially opposing trends are observed; however, increased overlap in the *ex vivo* condition corresponds to reduced separability.

### 3.3 Cancer versus Noncancerous Lifetime Comparison Across Tissue Types and Emission Wavelengths

To assess how fluorescence lifetime properties change between *in vivo* and *ex vivo* conditions, we compared the corresponding distributions within each tissue type, evaluating cancerous and noncancerous regions separately. In this context, separability reflects the distinction between *in vivo* and *ex vivo* measurements for each tissue category.

**Fig. 8:**
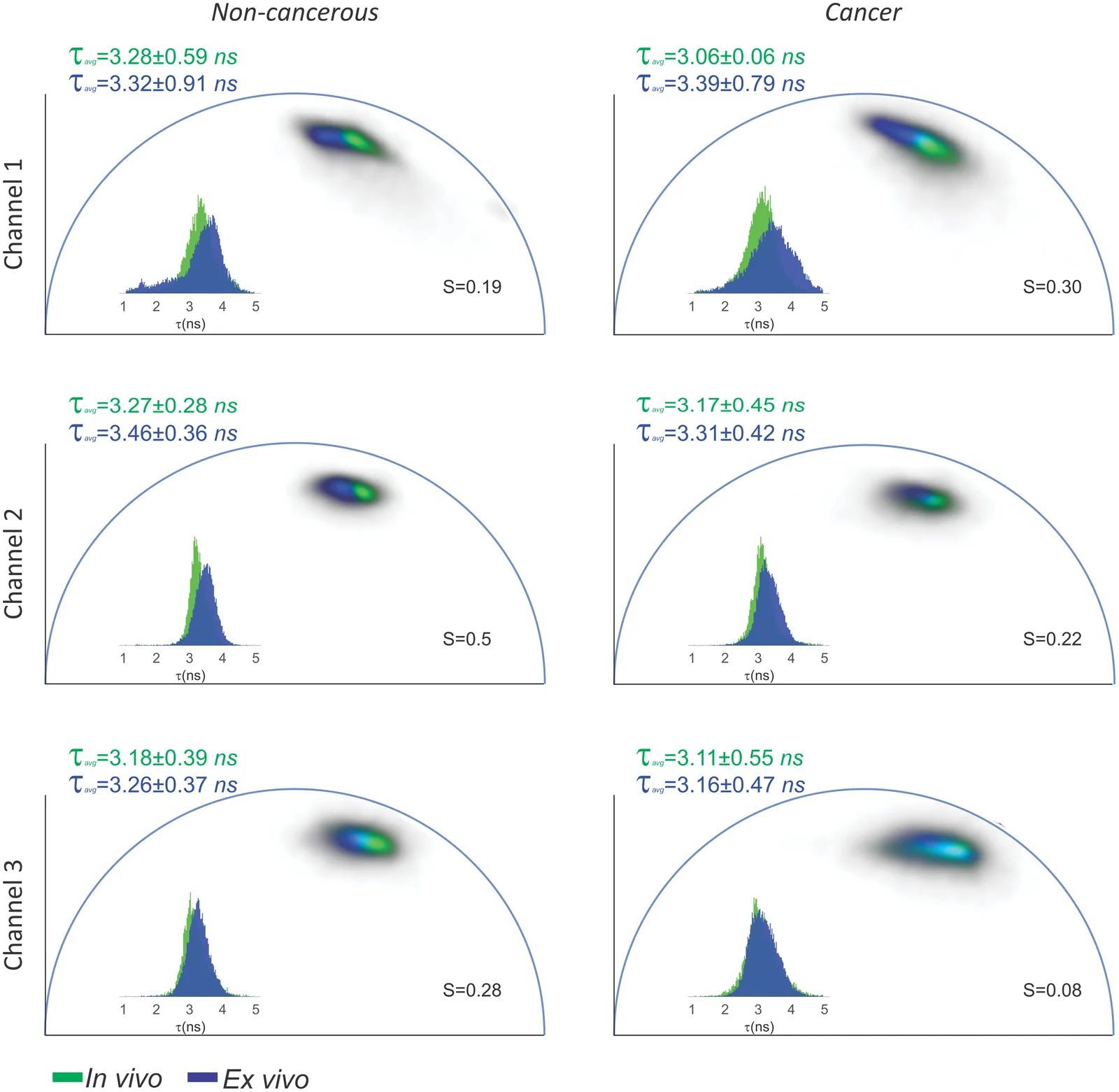
Phasor plots and histograms of average lifetimes for noncancerous and cancer tissue from oral tongue tissue highlight differences between *in vivo* and *ex vivo* FLIm measurements, labeled green and blue, respectively.

Figure 8 presents the separation between *in vivo* and *ex vivo* measurements for oral tongue tissue, evaluated independently for noncancerous and cancerous regions. In noncancerous tissue, separability varies across channels, with the highest separation observed in channel 2 (S=0.50), followed by channel 3 (S=0.28) and channel 1 (S=0.19). In contrast, cancerous tissue exhibits lower and more variable separability, with the highest value in channel 1 (S=0.30), decreasing in channel 2 (S=0.22) and reaching the lowest level in channel 3 (S=0.08). *In vivo*–*ex vivo* separability differs across tissue types and spectral channels, with noncancerous tissue showing higher separation in channel 2 and cancerous tissue showing the highest separation in channel 1.

**Fig. 9:**
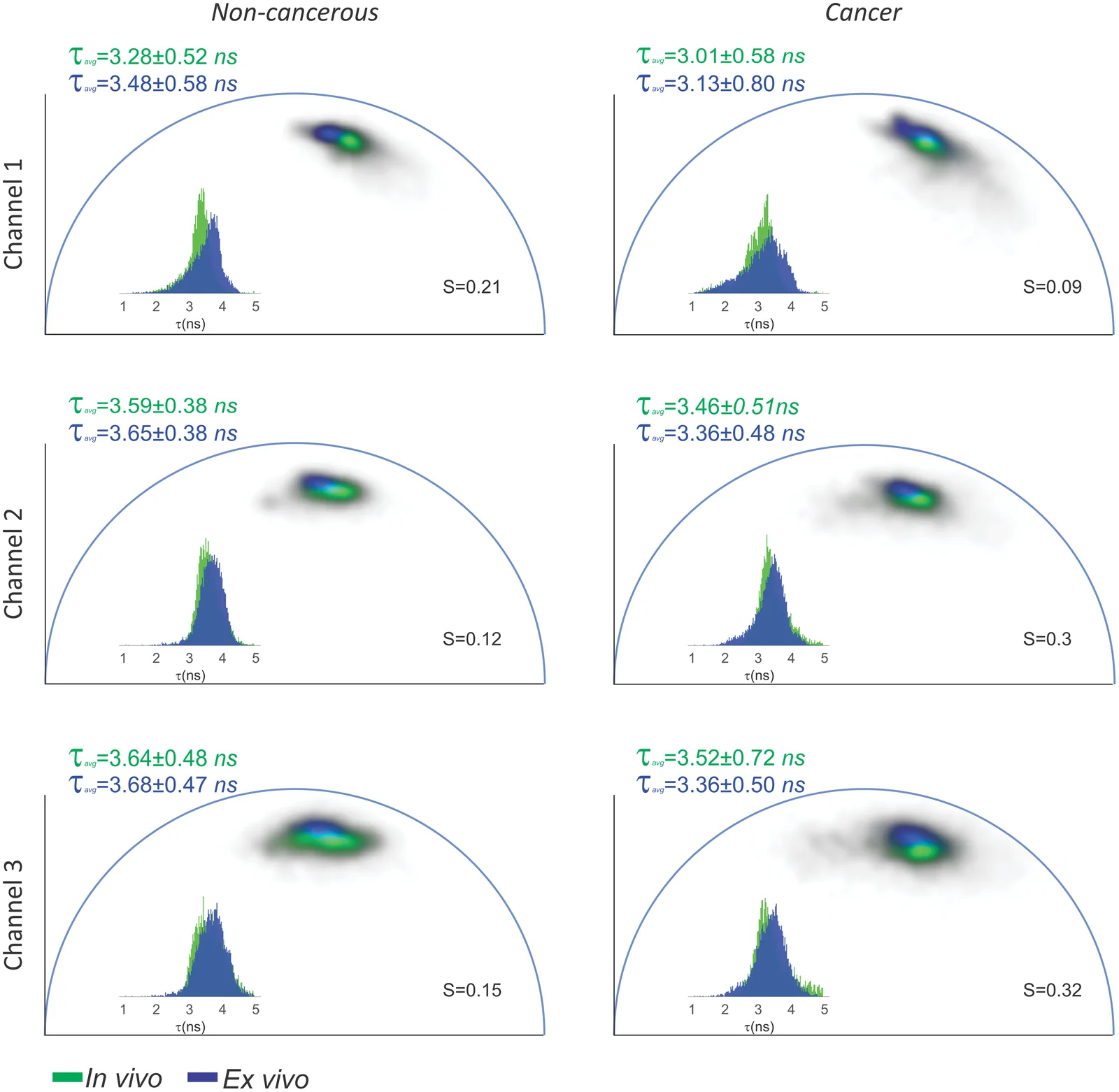
Phasor plots and histograms of average lifetimes for noncancerous and cancer tissue from the base of the tongue highlight differences between *in vivo* and *ex vivo* FLIm measurements, labeled green and blue, respectively.

Figure 9 shows the separation between *in vivo* and *ex vivo* measurements for base of tongue tissue. In noncancerous tissue, separability is low across all channels (S=0.21, 0.12, and 0.15 for channels 1 to 3). In contrast, cancerous tissue shows minimal separation in channel 1 (S=0.09) but higher separability in channels 2 and 3 (S=0.30 and 0.32), showing stronger distinction between conditions at longer wavelengths.

**Fig. 10:**
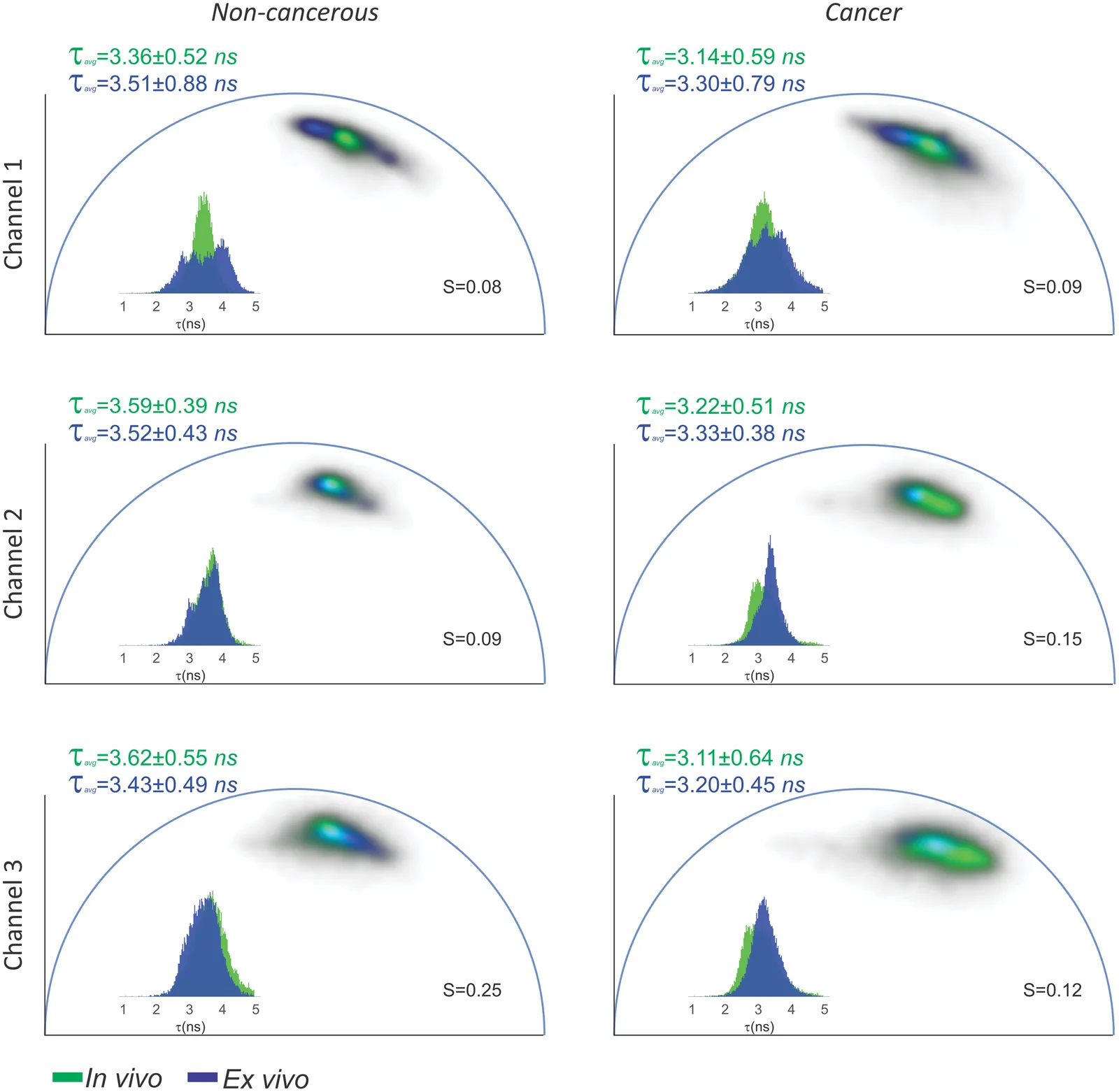
Phasor plots and histograms of average lifetimes for noncancerous and cancer tissue from tonsil highlight differences between *in vivo* and *ex vivo* FLIm measurements, labeled green and blue, respectively.

Figure 10 shows the separation between *in vivo* and *ex vivo* measurements for tonsil tissue. In noncancerous tissue, separability is low in channels 1 and 2 (S=0.08 and 0.09) and higher in channel 3 (S=0.25). In cancerous tissue, separability remains low to moderate across channels (S=0.09, 0.15, and 0.12), indicating limited distinction between conditions.

**Fig. 11:**
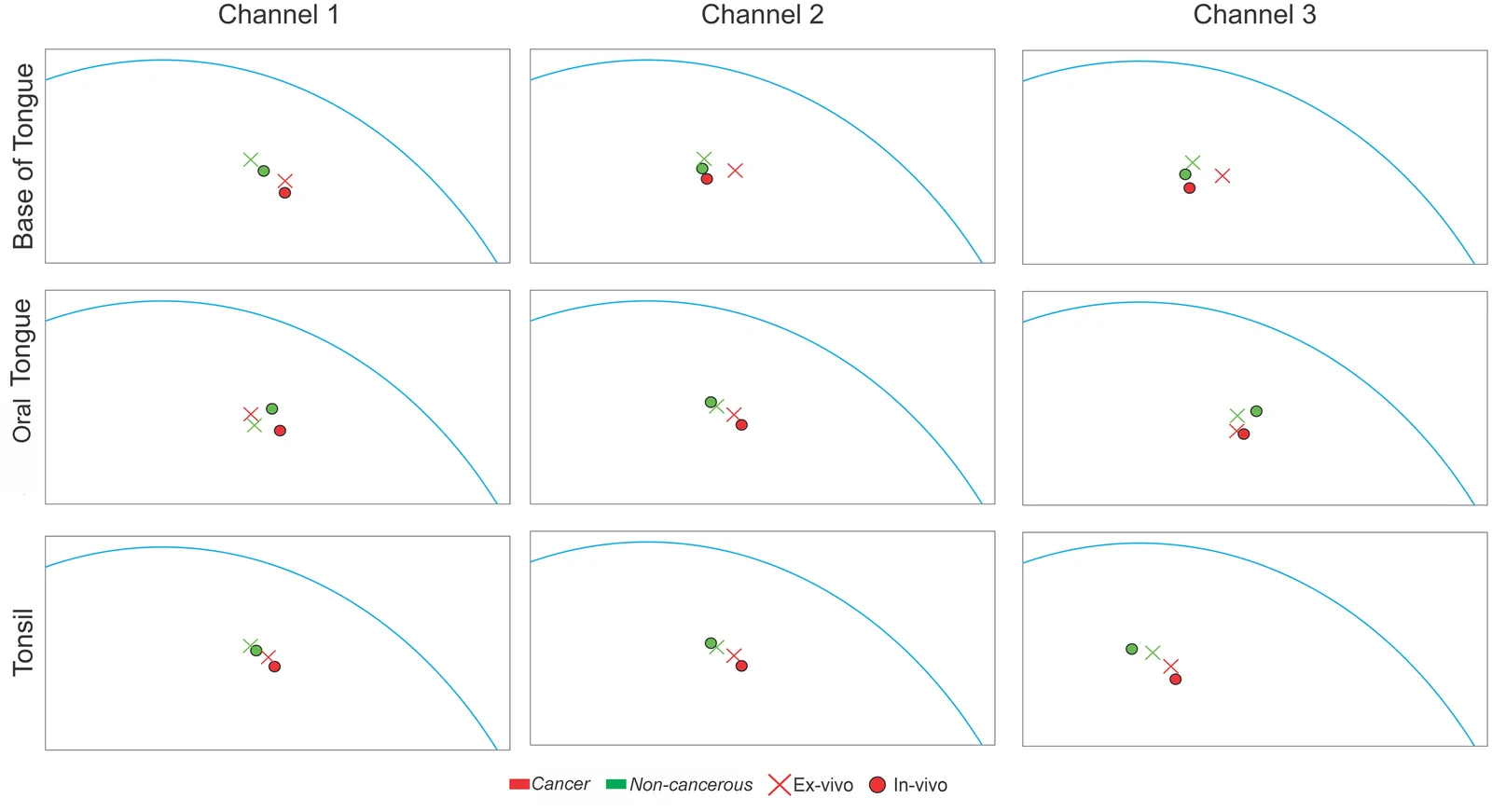
Phasor plot of the center of phasor clouds for all the tissue types. The separability index is represented by the distance between green and red symbols, with cancerous tissue shifting toward shorter lifetimes *in vivo*. The color green represents noncancerous tissue, whereas red represents cancerous tissue. In addition, X symbols represent *ex vivo* data, whereas O symbols represent *in vivo* data.

Figure 11 summarizes the centroid positions of phasor distributions derived from the datasets presented in Figs. 5–7, integrating all tissue types (oral tongue, base of tongue, and tonsil) and conditions (*in vivo* and *ex vivo*) into a single representation. Each point corresponds to the average phasor location for a given tissue type and condition, with O symbols indicating *in vivo* measurements and X symbols indicating *ex vivo* measurements, and colors distinguishing cancerous (red) and noncancerous (green) tissue.

Across tissues, distinct patterns emerge. In oral tongue and tonsil, greater separation between cancerous and noncancerous centroids is observed under *in vivo* conditions, particularly in channels 2 and 3. In contrast, the base of the tongue tissue shows greater separation in the *ex vivo* condition. In addition, cancerous tissue consistently shifts toward shorter lifetimes in the *in vivo* state relative to *ex vivo*, as reflected by the position of the red markers. These observations are consistent with the separability indices summarized in Table 2, where the base of the tongue tissue exhibits higher separability *ex vivo*, while oral tongue and tonsil show higher separability *in vivo* across most channels. These centroid relationships summarize how tissue type and measurement condition influence fluorescence lifetime separation.

**Table 2:** Separability indices for the base of the tongue, the oral tongue, and the tonsil.

	Channel 1		Channel 2		Channel 3	
	*In Vivo*	*Ex Vivo*	*In Vivo*	*Ex Vivo*	*In Vivo*	*Ex Vivo*
Base of tongue	0.33	0.38	0.15	0.43	0.13	0.39
Oral tongue	0.20	0.07	0.27	0.18	0.26	0.16
Tonsil	0.29	0.18	0.46	0.28	0.50	0.26

## 4 Discussion

Our results demonstrate that fluorescence lifetime signatures measured *in vivo* are not directly preserved following tissue resection and that the direction and magnitude of these changes are dependent on anatomical site. Although prior FLIm studies have focused primarily on *in vivo* tumor discrimination, our findings show that the transition to *ex vivo* conditions introduces tissue-specific shifts in fluorescence lifetime distributions that alter diagnostic separability. Notably, the direction and magnitude of fluorescence lifetime changes are not consistent across tissue types or spectral channels, indicating that no single transformation can reliably map *in vivo* measurements to *ex vivo* conditions. These findings indicate that fluorescence lifetime contrast is influenced not only by tissue properties but also by postresection physiological changes at the time of the imaging.

Previous FLIm studies have demonstrated notable discrimination between cancerous and noncancerous tissues *in vivo*; however, they have not fully addressed whether the same fluorescence lifetime signatures are maintained after tissue resection. In contrast, our results show that although cancerous and noncancerous tissues can still be distinguished *ex vivo*, the underlying lifetime distributions shift after resection. Specifically, *ex vivo* measurements exhibited broader histogram distributions compared to their *in vivo* counterparts. These broadened distributions likely reflect rapid physiological and biochemical changes following resection, including loss of perfusion, oxygen depletion, and disruption of cellular metabolism. However, the extent to which these changes impact separability remains dependent on tissue type.

The observed variability across anatomical sites likely arises from differences in tissue composition and microenvironment. For example, the base of the tongue contains a higher proportion of lymphoid and glandular tissue, whereas the oral tongue is more structurally dominated by muscle and stroma. These compositional differences, including variations in vascularity, perfusion, and tumor microenvironment conditions, influence the relative contributions of metabolic (NAD(P)H, FAD) and structural (collagen) fluorophores, resulting in distinct responses to ischemia and metabolic disruption following resection.^30^^,^^31^ These differences likely modulate the sensitivity of fluorescence lifetime signals to post-resection physiological changes.

Importantly, diagnostic performance is governed by the relative separation and overlap of fluorescence lifetime distributions rather than by differences in mean lifetime values alone. As observed in oral tongue tissue (Fig. 5), both cancerous and noncancerous regions exhibit increased average lifetimes in the *ex vivo* condition, and in some channels the difference between mean lifetimes even increases (e.g., channel 2). However, this is accompanied by broader distributions and increased overlap, resulting in reduced separability compared with the *in vivo* condition. This highlights a key limitation of relying solely on average lifetime metrics, as they do not capture the multiexponential nature of fluorescence decay or the distribution-level changes that determine diagnostic contrast. In contrast, phasor-based analysis more effectively reflects these differences by preserving the full distribution characteristics and enabling more accurate assessment of tissue separability.

Current findings also show that FLIm’s ability to discriminate between various tissue types differs between*in vivo* and *ex vivo* imaging, with oral tongue and tonsil showing higher contrast *in vivo*, while the base of the tongue demonstrates improved discrimination *ex vivo*. These results suggest that both tissue type and anatomical location are important considerations when determining the optimal utilization of FLIm, and they reinforce the value of incorporating both *in vivo* and *ex vivo* imaging strategies to leverage their respective strengths. This observation is further supported by the *in vivo*–*ex vivo* comparisons within each tissue type (Figs. 8–10), which show variable separability patterns across tissues and channels.

From a clinical perspective, these findings indicate that FLIm-based diagnostic approaches derived from *ex vivo* measurements may not directly translate to intraoperative *in vivo* use. This has important implications for machine learning-based classification, as each imaging condition exhibits distinct fluorescence lifetime characteristics that affect performance. Consequently, models developed using *ex vivo* data cannot be assumed to generalize to *in vivo* settings, and separate development and validation strategies may be required. Understanding the source of these diagnostic differences requires examining how fluorescence lifetime distributions and their associated separability differ between *in vivo* and *ex vivo* environments,^32^^,^^33^ as well as rapid postresection physiological alterations.^34^ Although FLIm *ex vivo* shows promise, deploying it clinically will require addressing the unique challenges of *ex vivo* imaging workflows. In this context, standardizing acquisition timing and developing analysis methods robust to these variations may improve reproducibility and facilitate translation.

Integrating FLIm into the surgical workflow requires navigating the distinct advantages and limitations inherent to both *in vivo* and ex vivo imaging environments.^14^^,^^35^ An advantage of ex vivo imaging is the ability to use higher excitation light intensity and longer acquisition times, resulting in improved signal-to-noise ratios. In addition, ex vivo imaging allows for all sides of the tissue to be assessed, allowing for a comprehensive assessment of the excised specimen’s surface. This complements *in vivo* measurements, which can be used in assessing deep margins and directly identifying residual tumor within the surgical cavity.^36^ Although the ex vivo format improves correlation with downstream histology, technical challenges must be addressed to accurately map positive margin findings back to the surgical site. A combined workflow leveraging both imaging conditions may provide a more comprehensive intraoperative assessment.

This study was designed to integrate seamlessly with the clinical workflow by acquiring paired *in vivo* and immediate ex vivo FLIm measurements following specimen excision. Consequently, the interval between measurements varied across cases, reflecting routine surgical and specimen-handling procedures. In addition, only the initial ex vivo measurement acquired in the operating room prior to transfer to the histopathology laboratory was included. Although this design enabled clinically relevant comparisons between *in vivo* and immediate ex vivo measurements, it did not allow assessment of the temporal evolution of fluorescence lifetime following tissue resection. Although Channel 4 may provide biologically relevant information related to porphyrins and tissue oxygenation, its fluorescence signal was insufficient for reliable lifetime analysis in the present study. Also, certain anatomical sites had limited representation of noncancerous tissue, and the sample size within each anatomical subgroup remains limited, which may affect the generalizability of tissue-specific observations and may affect the robustness of separability estimates. Finally, the study is limited to head and neck cancers, and further validation across additional tissue types is required.^11^^,^^18^

Future work should therefore focus on both expanding the range of tissues studied and characterizing the temporal evolution of fluorescence lifetime changes immediately following tissue resection, which may improve modeling of ex vivo signal dynamics. In addition, incorporating multi-channel and spatial features into analysis frameworks may enhance diagnostic robustness and support improved generalization across imaging conditions. These efforts will be critical to effectively integrate FLIm into both surgical guidance and pathology workflows.

## 5 Conclusion

This study demonstrates that fluorescence lifetime properties are altered by tissue resection in a tissue- and wavelength-dependent manner. These changes directly impact diagnostic separability and highlight the need for condition-specific interpretation of FLIm data. Together, these findings demonstrate that fluorescence lifetime contrast depends on the physiological state of the tissue at the time of the imaging, rather than being determined solely by intrinsic tissue properties, emphasizing the importance of context-specific interpretation for the reliable clinical translation of FLIm.

## Data Availability

De-identified data may be shared with qualified investigators subject to approval by the UC Davis Institutional Review Board and execution of any required data use agreements. Requests should be directed to the corresponding author and will be reviewed in accordance with institutional policies.
